# The Role of Artificial Intelligence in Managing Multimorbidity and Cancer

**DOI:** 10.3390/jpm11040314

**Published:** 2021-04-19

**Authors:** Alfredo Cesario, Marika D’Oria, Riccardo Calvani, Anna Picca, Antonella Pietragalla, Domenica Lorusso, Gennaro Daniele, Franziska Michaela Lohmeyer, Luca Boldrini, Vincenzo Valentini, Roberto Bernabei, Charles Auffray, Giovanni Scambia

**Affiliations:** 1 Scientific Directorate, Fondazione Policlinico Universitario A. Gemelli IRCCS, 00168 Rome, Italy; alfredo.cesario@policlinicogemelli.it (A.C.); antonella.pietragalla@policlinicogemelli.it (A.P.); domenica.lorusso@policlinicogemelli.it (D.L.); gennaro.daniele@policlinicogemelli.it (G.D.); franziskamichaela.lohmeyer@policlinicogemelli.it (F.M.L.); giovanni.scambia@policlinicogemelli.it (G.S.); 2 Department of Ageing, Neurosciences, Head-Neck and Orthopaedics Sciences, Fondazione Policlinico Universitario A. Gemelli IRCCS, 00168 Rome, Italy; riccardo.calvani@guest.policlinicogemelli.it (R.C.); anna.picca@guest.policlinicogemelli.it (A.P.); roberto.bernabei@policlinicogemelli.it (R.B.); 3 Gynecological Oncology Unit, Fondazione Policlinico Universitario A. Gemelli IRCCS, 00168 Rome, Italy; 4 Department of Life Sciences and Public Health, Faculty of Medicine and Surgery, Università Cattolica del Sacro Cuore, 00168 Rome, Italy; 5 Radiation Oncology Unit, Fondazione Policlinico Universitario A. Gemelli IRCCS, 00168 Rome, Italy; luca.boldrini@policlinicogemelli.it (L.B.); vincenzo.valentini@policlinicogemelli.it (V.V.); 6 European Institute for Systems Biology and Medicine (EISBM), 69390 Vourles, France; cauffray@eisbm.org

**Keywords:** personalized medicine, artificial intelligence, omics, geriatrics, multimorbidity, gynecological oncology, oncology, deep learning, machine learning, internet of things

## Abstract

Traditional healthcare paradigms rely on the disease-centered approach aiming at reducing human nature by discovering specific drivers and biomarkers that cause the advent and progression of diseases. This reductive approach is not always suitable to understand and manage complex conditions, such as multimorbidity and cancer. Multimorbidity requires considering heterogeneous data to tailor preventing and targeting interventions. Personalized Medicine represents an innovative approach to address the care needs of multimorbid patients considering relevant patient characteristics, such as lifestyle and individual preferences, in opposition to the more traditional “one-size-fits-all” strategy focused on interventions designed at the population level. Integration of omic (e.g., genomics) and non-strictly medical (e.g., lifestyle, the exposome) data is necessary to understand patients’ complexity. Artificial Intelligence can help integrate and manage heterogeneous data through advanced machine learning and bioinformatics algorithms to define the best treatment for each patient with multimorbidity and cancer. The experience of an Italian research hospital, leader in the field of oncology, may help to understand the multifaceted issue of managing multimorbidity and cancer in the framework of Personalized Medicine.

## 1. Introduction

**Figure 1:**
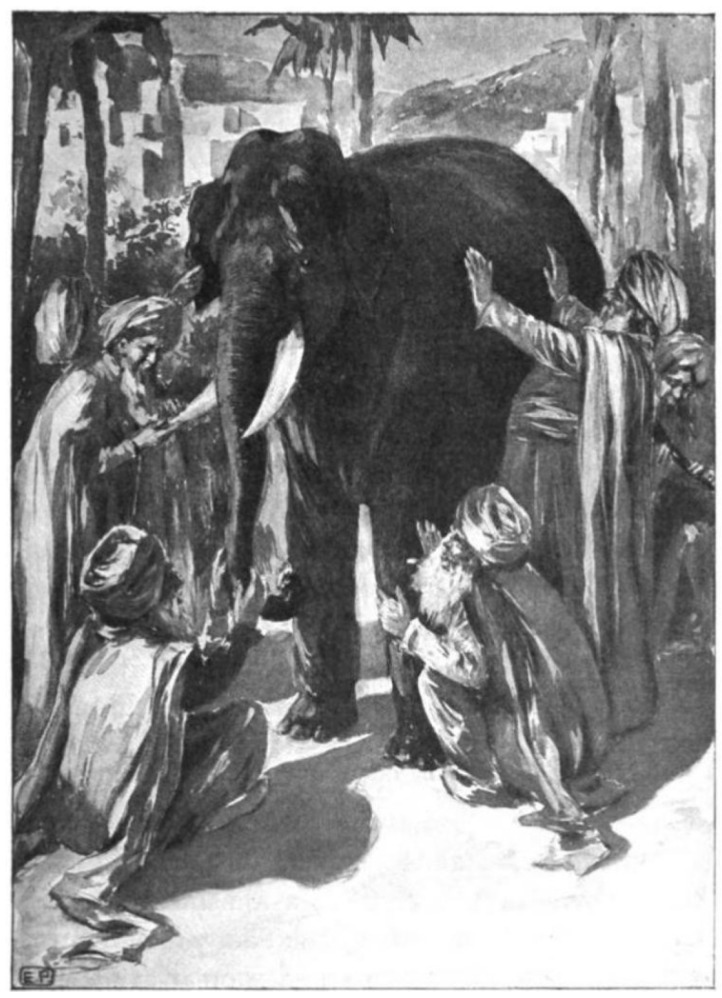
By illustrator unknown from *The Heath readers by grades*, D.C. Heath and Company (Boston), p. 69, Public Domain [1].

In a famous Indian parable, a group of blind men encountered an elephant and tried to learn what it is like by touching a single different part of the animal. Inevitably, they ended up disagreeing on their guess. Indeed, they were unable to capture the whole picture and trusted only their partial experience what they considered as the only possible truth (Figure 1).

Traditional healthcare paradigms are built on a similar reductionist view of the patient as an individual affected by a single condition (or a sum of conditions). In the 21st century, the biomedical framework has moved away from a disease-centered model (that focused on individual organs, tissues, cells, or molecules) to a more personalized approach [2]. Personalized Medicine (PM) represents an innovative approach to address care needs of vulnerable populations. The promise of delivering “the right treatments, at the right time, every time to the right person” was nurtured by the availability of sophisticated omics (e.g., genomics, proteomics, metabolomics, radiomics) platforms that allowed to investigate the biological features of a person with unprecedented details [3]. In some cases, PM tends to reduce patients to a collection of unique biomarkers detailing phenotyping; this reductionism is not always suitable for complex conditions. Whereas some disease-modifying genes contribute to phenotype expression, other factors such as the exposome (e.g., nutrition, the environment, and lifestyle) can affect disease progression [2].

In addition to these data sources, researchers are now investigating the possible added value coming from the introduction of Real-World Data (RWD), real-life biomedical data recorded in registries, clinical records, biobanks, administrative or insurance databases or harvested through surveys or mobile applications.

At a broader level, personalized approaches consider other relevant characteristics such as lifestyle and individual preferences, which is in opposition to the more traditional “one-size-fits-all” strategy focused on interventions and care services designed at the population level [4]. First, PM has been successfully applied to the treatment of mono-factorial diseases in which a single and specific disease predictor is identified and targeted by the medical intervention. Interestingly, artificial intelligence (AI) has been successfully applied also to standard diagnostic bioimaging, achieving the possibility of extracting quantitative features by means of dedicated machine learning and bioinformatic software and using them to set up both predictive and characterization models through the so-called radiomics approach [5,6,7].

The demographic transition industrialized countries have experienced in the last decades has prompted a reshaping of the way patient needs should be addressed. Today, the typical patient accessing healthcare services is an older adult with multiple morbidities, often with functional impairment and unmet medical needs [8,9].

Multimorbidity is a health condition transversal to every medical field and heterogeneous in its clinical manifestations with variable impact on quality of life, which makes the management of contemporary geriatric and oncological patients one of the most challenging tasks of modern medicine [10,11]. Approximately 70% of people above 60 years match this identikit, which makes multimorbidity the most prevalent chronic condition [8].

Multimorbidity prevalence progressively increases with age, affecting more than 60% of people over 65 years but can also occur in younger ages, especially if related to a low socioeconomic status [3,4]. Currently, in developed countries, owing to improvements in health care and nutrition, a gradual increase of older patients with multiple chronic conditions, including cancer, has been recorded. Moreover, as survivorship increases, more patients will be living with long-term consequences that affect health and quality of life. In 2019, 20.3% of the total European population was aged over 65 years and it is expected that by 2100 the population over 65 years will account for 31.3% [12]. It is estimated that 50% of all cancer cases are diagnosed in patients older than 65 years [13].

Multimorbidity itself is intrinsically complex. Its onset, progression, and severity result from combined (and often synergistic) effects of clinical, behavioral, environmental and lifestyle factors, revealing its intrinsically complex nature [14,15]. A further level of complexity arises from the timeframe during which those factors interact, which may span over decades and with different contributions of single factors across each life stage [11]. It is therefore crucial to understand how granular data can be integrated with the “wholeness” of the patient, taking advantage of the advanced AI based data retrieval tools currently available for the different omics domains and the immense assets of RWD, which represent an innovative and promising approach able to integrate traditional clinical decision and research paradigms.

## 2. Multimorbidity in Geriatrics and Oncology

A true implementation of the PM paradigm of care for older adults with multimorbidity will revolutionize the current treatment of chronic diseases, which is still shaped on the notion of an ‘average person’ within large populations, with little consideration for individual differences and burdened by significant data loss and under-representation. However, major obstacles need to be overcome for the application of PM approaches in the geriatric multimorbid framework.

In the geriatric field, especially when dealing with multimorbidity, a multidimensional approach is mandatory. Modern geriatrics have been built around a new generation of comprehensive geriatric assessment (CGA) instruments, each designed for specific healthcare settings and addressing the multifaceted issues of the older patient [16]. CGA tools go beyond the omics make-up of a person and enable a global appraisal of the individual, taking into consideration at the same time their clinical, functional, and socioeconomic characteristics. The output of CGA is, therefore, the design of a true personalized geriatric care plan tailored to an older person’s needs, successfully integrating several different data sources [16].

The robustness of CGA-based patient management was highlighted during the 2020 American Society of Clinical Oncology (ASCO) Annual Meeting in which several high-impact studies were presented showing remarkable results obtained through the implementation of CGA–guided interventions in older adults with cancer [17]. Four major randomized controlled trials showed improvements in established oncologic outcomes, including drug toxicity, quality of life, and survival in older adults with solid and hematologic malignancies who received an integrated oncogeriatric management, confirming the need of a holistic approach, rather than a sub-specialistic one [18].

The recognition by the American Society of Clinical Oncology (ASCO) together with efforts from major international societies of geriatric oncology supports the integration of CGA and CGA-based interventions into routine oncologic care to offer people with cancer a multidisciplinary individualized care plan [19]. Digital health or digital solutions, such as AI, have been successfully developed for decision making, symptom management, and follow-up monitoring and therefore, are increasingly important for cancer survivors and their caregivers [20,21].

## 3. The Example of Gynecological Oncology

Cancer is considered a chronic and complex disease which can be controlled and managed with integrated treatments, if necessary, over long periods spanning years or decades. Due to the complexity of the oncological clinical scenario, cancer phenotypes are not always attributable to one or more omic drivers. Moreover, the co-existence of two or more chronic conditions including mental and physical conditions defined as multimorbidity is the most common chronic condition and represents an increasingly frequent condition even in patients with cancer [22].

Evidently, the coexistence of cancer in older age patients with other chronic conditions has significant implications for cancer screening, treatment choice and outcomes for both cancer and chronic diseases [23]. As for other neoplasms, as well as for gynecological cancer, multimorbidity affects patients beginning from the diagnosis to therapeutic and screening stages; it is therefore crucial to optimize patient management for a successful implementation of PM. The identification and appropriate management of multimorbidity effects in patients with cancer is an increasing issue not only for oncologists but also for other clinicians involved in patient treatment.

The complexity of cancer and multimorbidity at the individual level make decision-making and the management of different information difficult. Patient Reported Outcomes (PROs) and electronic health records aiming at discovering patient-specific patterns of disease progression and providing real-time decisions represent innovative and promising RWD sources that may be successfully integrated also in the PM research perspective [24].

Moreover, among gynecological cancer patients, the clinical scenario of multimorbidity is heterogeneous and it is characterized by high managing complexity and frailty, which may influence diagnostic work-up, treatment efficacy, and patient survival. Gynecological neoplasms include ovarian, uterine (endometrial, cervical cancer and uterine sarcoma) vaginal, and vulvar cancers, which accounted for 11.3% of new female cancer cases in 2018. Usually, gynecological cancers are described together, but each malignancy is associated with distinct risk factors, signs, symptoms, prognosis, and specific treatments. Ovarian cancer (OC) and endometrial cancer (EC) arise in postmenopausal women on average in the sixth life decade whereas cervical cancer occurs typically in younger women. Patient survival rates vary across different cancer types and are largely dependent on the stage at diagnosis [25].

Overall, limited data for cancer management in patients with multimorbidity are available and no ideal approach to measure comorbidity in the context of cancer has been proposed. One of the suggested methods to evaluate comorbidity is the Charlson Comorbidity Index based on the International Classification of Diseases (ICD), which is an index that combines the number and severity of concomitant diseases.

There are some reasons why gynecological cancer and multimorbidity can coexist beyond older age. Environmental risk factors such as smoking, poor diet, obesity, diabetes, lack of physical activity, alcohol abuse, chronic infections (Human papillomavirus [HPV]) are risk factors both for cancers and chronic conditions. For example, HPV infection has been implicated in 99.7% of cervical squamous cell cancers, while 40% of endometrial cancers are directly related to obesity [26]. Patients with multimorbidity may experience both a delay in diagnosis or—due to a close relationship with the health care system—they may be diagnosed in an earlier stage. Therefore, several data should be integrated to personalize diagnosis and treatment of each specific patient, taking into account all the necessary data sources.

## 4. Results

**Figure 2:**
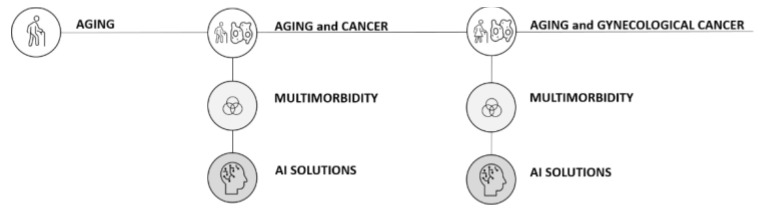
Visual representation of the literature. AGING: [16,17,27,28]. AGING and CANCER: [16,17,18,26,29]; MULTIMORBIDITY: [15,27,30,31,32,33,34,35,36,37,38,39,40,41]; AI SOLUTIONS: [20,21,42,43,44,45,46,47,48]. AGING and GYNECOLOGICAL CANCER: [13,49,50,51,52,53,54]; MULTIMORBIDITY: [22,23,25,49,50,53,55,56]; AI SOLUTIONS: [24,57,58].

Continuous research is fundamental to understand the possibilities of intervention in the complex scenarios of multimorbidity and oncology. When applied into practice, PM embraces each stage of the clinical path towards the identification and selection of specific characteristics and response to treatments. The fusion of the state of the art on this topic (Figure 2) with the experience on the field from our institution Fondazione Policlinico Gemelli in Rome (which has been recognized in 2018 as research hospital for “Personalized Medicine” and “Innovative Biotechnologies” by the Italian Ministry of Health) highlights the advantages of PM, without neglecting the challenge of managing data and the integration of them in patients’ daily lives.

### 4.1. Clustering Chronic Diseases

Our Oncogeriatric Unit implemented an innovative paradigm of care based on the combination of CGA with the longitudinal assessment of biological and functional biomarkers of ageing [29]. This approach is based on the integration of the multidomain nature of CGA with the new concept of geroscience, according to which perturbations in specific molecular pathways or “hallmarks of aging” are responsible for the development of age-related conditions [26,27]. The aim of this new care model is to capture elements (and associated biomarkers) which reflect age-related derangements in physiological systems with the objective to develop interventions to manage multimorbidity and to improve physical function and overall quality of life of older persons with cancer [28]. Similar approaches are also underway in other geriatric settings to identify predictors and to implement interventions against neurodegenerative diseases [59,60] and other age-related conditions, such as physical frailty and sarcopenia (PF&S) [61].

PF&S was operationalized by our Geriatric Team in the context of the «Sarcopenia and Physical fRailty IN older people: multi-componenT Treatment strategies» (SPRINTT) project, one of the largest geriatric initiatives ever conducted in Europe [30]. SPRINTT was conceptualized to identify “real life” vulnerable older person at risk of mobility disability and to promote the implementation of successful ageing strategies across Europe through the adoption of personalized intervention protocols [31]. The key elements of the novel nosological entity of PF&S are the presence of a target organ damage (i.e., low skeletal muscle mass) and clinical manifestations pertaining to the physical function domain (i.e., weakness, slow walking speed, and poor balance), which can be objectively assessed using the Short Physical Performance Battery (SPPB) [32].

It is worth mentioning that the European Medicines Agency (EMA) recently identified the SPPB as the preferred option to characterize physical function in clinical trials in vulnerable geriatric patients at risk of adverse outcomes [33]. This choice was based on its “prognostic value of disability and mortality; validation status; feasibility of use across all therapeutic areas; ease of use; time required; ease of investigator’s training; cost” making the SPPB a full-fledged functional biomarker of ageing. Notably, the SPPB and other physical performance tests, such as habitual gait speed, are increasingly used in the field of geriatric oncology and cardiac surgery to assist care individualization and prognostication [34,35]. Gait speed may also be used as a simple clinical prognostic (bio)marker in older adults with cardiovascular and neuropsychiatric multimorbidity [36].

Given the complex nature of multimorbidity, a possible solution to foster PM approaches may be to reduce dimensionality by focusing on clusters of chronic diseases [37]. Diseases tend to cluster together due to common risk factors, shared pathophysiology, or causative links (i.e., one disease can directly cause another) [37]. Diseases that co-occur in the same individual beyond chance can be defined through this approach, helping to identify the underlying factors and develop preventive and/or therapeutic interventions targeting common pathways. The concept of disease clustering may be combined with PM precepts through the collection and analysis of large datasets of epidemiological, biological and RWD.

Machine Learning (ML) algorithms can help identifying clinically meaningful patterns by analyzing RWD (i.e., demographics, ICD v. 10 chronic diagnoses, prescribed drugs, and socio-economic status) from elderly populations (≥65 years) [42] to analyze the trajectories of mortality [43]. Genetic predisposition and blood-derived determinants should be investigated together with other well-established personal and contextual determinants of multimorbidity (i.e., sex, social and living environments, education, and physical function parameters) to design strategies to comprehensively target multimorbidity [27]. While the definition of molecular/genetic targets for multimorbidity is still in its infancy, a consensus panel of geriatricians and gerontologists recently proposed a conceptual framework to select circulating biomarkers to be used in “geroscience-guided” clinical trials [38]. A short list of blood-based biomarkers was suggested and the first trials testing drugs targeting age-related disease mechanisms are currently ongoing [39,40].

According to Zhavoronkov et al., Deep Learning (DL) systems, trained on measurable features changing in time (deep aging clocks), can help aging research by establishing causal relationships in non-linear complex systems. These predictive models can anticipate health trajectories or mortality, and support identification and tailoring of novel therapeutic targets, representing since long time a powerful resource also in several fields of clinical and translational oncology [45,46].

Our Department of Geriatrics and Gerontology is actively collaborating with the Aging Research Center at Karolinksa Institutet to unveil the complex interplay among biological, personal, and environmental factors in shaping clinical trajectories of multimorbidity in older adults [36,41]. The implementation of digital health technologies may also be part of a PM approach for elderly with multimorbidity.

The World Health Organization (WHO) launched the Integrated Care for Older People (ICOPE) tool package to assist healthcare and social professionals to improve care of the older adults [46]. Moreover, wearable devices and integrated systems, such as smart homes, which incorporate environmental and medical sensors (the so called “Internet of Things”), and modern information and communication technologies (ICT), may allow the continuous and remote monitoring of health status and wellbeing of older people at acceptable costs [47]. With the increasing digital literacy of baby boomers, older adults will be actively involved in decisions about their treatment options. Indeed, blockbuster devices, such as smartphone, which are increasingly used by older people, may provide an excellent means for continuous data collection and assessment of PROs [48].

### 4.2. Managing Multimorbidity for Gyneco-Oncological Patients

As mentioned above, multimorbidity may significantly affect gynecological cancer patients. The prevalence of gynecological cancer comorbidity is not well characterized but it is estimated that about four out of ten patients with cancer have at least one chronic condition and 15% of patients with cancer have at least two or more chronic conditions (cardiovascular, metabolic illness, obesity, mental health, and musculoskeletal conditions) [55]. Moreover, patients with comorbidity and older patients are less likely to receive treatment with curative intent and are often excluded from clinical trials and aggressive therapeutic strategies that could potentially be more efficacious [62].

OC represents a complex model in which genomic factors (DNA Homologous recombination repair system) are integrated in the choice of treatment (surgery and chemotherapy); in this setting, multimorbidity could influence optimal treatment strategies constituting a significant stressor [56]. Moreover, despite the recent development of new targeted therapies such as bevacizumab and Poly ADP-Ribose Polymerase inhibitors (PARP-I), OC prognosis remains poor, with a five-year survival rate of 43% overall and 25% for women aged >75 years [13]. Data from the Danish cancer registry on 5213 OC patients indicate that the presence of severe comorbidity was associated not only with advanced OC stage at diagnosis but also with increased mortality among patients with comorbidities in which the impact of comorbidity varied by stage [49]. Moreover, an epidemiological study conducted by Wright et al. on 49,932 OC patients diagnosed from 1975 to 2011, showed that in women with advanced stage (FIGO III and IV), survival decreased with increasing age [50].

When OC is diagnosed, the risk for postoperative complications should be recognized preoperatively to personalize the optimal surgical strategy (i.e., primary debulking surgery versus neoadjuvant chemotherapy). The prediction of postoperative complications at primary debulking surgery allows clinicians to achieve more objective decisions regarding the personalized strategy for primary treatment in advanced OC.

Patients surgically treated for ovarian cancer may be readmitted for postoperative complications after hospital discharge, which could have been prevented with monitoring; web-based apps can collect real-time postoperative health care information, which are integrated in electronic health records to monitor patients after their discharge [57]. Vizzielli et al. developed and validated in our institution a simple prognostic laparoscopic score to predict postoperative complications in advanced OC patients undergoing primary debulking surgery. The predictive score is based on the following variables: poor performance status, presence of ascites (>500 cm^3^), CA125 serum level (>1000 U/mL), and high laparoscopic tumor load (predictive index value, PIV ≥ 8). The mean risk of developing major postoperative complications was 3.7% in patients with scores from 0 to 2, 13.2% in patients with scores from 3 to 5, 37.1% in patients with scores from 6 to 8. This simple predictive score can be used as a preoperative tool to adopt therapeutic strategies tailored on an individual basis in accordance with patient’s ability to tolerate treatment, regardless of age. A Vizzielli’s score calculator is available as a web app, through which surgeon may accurately predict patients’ postoperative outcome by early identifying high-risk woman, thus adopting tailored strategies on individual basis.

Generally, patients with comorbidity received fewer curative strategies [58]. In a population-based study, Maas et al. evaluated the influence of age and co-morbidity on treatment and prognosis in 1116 OC patients. The authors showed that the prevalence of co-morbidity was 63% for the age group >70 versus 34% of the younger age group. More than 80% of patients with advanced OC younger than 70 years underwent the recommended therapeutic strategy (surgery and chemotherapy) compared to only 45% of patients aged 70 or older. Mortality was higher among patients with comorbidities and the impact of comorbidity varied by stage.

Wright et al. showed on 9587 elderly patients with stage II-IV OC that the use of primary surgery decreased from 63.2 to 49.5%, while primary chemotherapy increased from 19.7 to 31.8% from 1991 to 2007 (*p* < 0.0001) without difference in terms of survival [51]. Fanfani et al. also demonstrated that elderly (65–75 years) and very elderly (>75 years) patients might tolerate radical and ultra-radical surgery without an increase of morbidity and with clinical outcomes similar to those reported in younger cases [52]. After surgery, multimorbidity, in particular in elderly patients, can affect the choice of medical treatment. Often, elderly or frail women do not receive standard chemotherapy regimens compared with younger patients due to poor physical or cognitive performance status and the risk of mortality. However, chronological age as the only factor should not be used to guide treatment decisions, as it does not consider frailty.

Clinicians seem to be unprepared to treat elderly patients, and many data indicate that numerous patients are under-treated because of the fear of intolerable side effects, thus limiting their possibility of satisfactory treatments and survival. Three-weekly platinum-based chemotherapy is considered the standard chemotherapy regimen for advanced OC. However, weekly administration of carboplatin-paclitaxel seems to be safe also in patients with multiple comorbidities. A multicentric Italian study by the MITO group describes safety of a weekly treatment with carboplatin (AUC 2) and paclitaxel (60 mg/m^2^) in patients with OC over 70 years of age. The population includes more than 50% of patients with two or more comorbidities (hypertension, diabetes, osteoporosis, and arthrosis). The study demonstrated the feasibility of weekly platinum-based chemotherapy with a favorable toxicity profile [53].

A French retrospective study including 147 women ≥70 years treated for OC between 2007 and 2015 was conducted to investigate the surgical approach in elderly women and to compare the effects of age and frailty on surgical procedures, complications, and prognosis. The authors showed that 77% of the 70–74 age group received optimal treatment compared with 51% of women older than ≥75 years (*p* = 0.018). The older group experienced fewer postoperative complications (22.6% vs. 38.9%, *p* < 0.001) versus 70–74 age group due to a less aggressive surgical approach to reduce immediate postoperative complications (32% of bowel resections versus 67%, *p* < 0.001). Regarding medical treatment older women also received more chemotherapy with platinum only (15% vs. 2%, *p* = 0.007) and less bevacizumab (9% vs. 32%, *p* = 0.003). Frail patients with a modified Charlson Comorbidity Index score > 3 had a five-year survival rate of 30% versus 62% for those with a score < 3 (*p* < 0.001) [54]. Moreover, no prospective studies evaluated the effect of chronological age and multimorbidity on surgical approaches, postoperative complications, and medical treatment.

Furthermore, in addition to constitutive variables, the genetic profile can change during disease progression. The ability of personalizing therapeutic combinations according to the complexity of each specific person is going therefore beyond the abilities of the individual physician. AI solutions have been developed to support the human operators in their collegial decisional process [63].

## 5. Discussion and Conclusions

The access to powerful technologies for extracting, analyzing, and managing heterogeneous medical records coupled with patient lifestyle information, will boost the PM paradigm-shift with significant ethical and societal implications [64]. Tailored AI solutions may help to treat amounts of mixed data [65]. For example, ML algorithms can find hidden patterns in data, classify them according to their characteristics, and then match patients/diseases/drugs based on their common features [66]. These algorithms may open new venues to unravel the complexity of multimorbidity to identify the most relevant factors involved in the onset, progression, and clustering of chronic diseases. This information may be successfully used to identify high-risk individuals and improve their clinical management [67].

PM may successfully support innovative approaches to achieve individual treatment of frail patient presenting multimorbidity. The technological advances of the last decades have spurred the interest around the implementation of PM going beyond the boundaries of research and entering in daily clinical practice trough AI based decisional support systems. This may represent an epochal change in the care of multimorbidity. In order for that approach to be fully developed and made broadly available, the PM scope should be enlarged to include personal factors, such as education, lifestyle, physical function, environmental and social elements, and individual preferences. The omics revolution enabled characterizing biological details, which will be relevant to prevent and treat most chronic diseases and multimorbidities. To avoid “the blind men and the elephant” fallacy, the way to tackle complexity in multimorbidity should not simply be the sum of multiple reductionist characterizations (i.e., the sum of single omics determinations). It is now clear that the “intuitive” approach to cancer patient with multimorbidity may results in a sub-optimal patient treatment. Usually, cancer treatment guidelines are primarily based on the results of clinical trials from which patients with comorbidity and/or older age are often excluded; patients with multimorbidity should be routinely enrolled in clinical trials and research on real-world evidence (data collected outside of conventional RCTs) encouraged to enhance the scientific value of these observations.

In conclusion, a new forma mentis is required to provide personalized care and tailor the most appropriate surgical and medical treatment for a frail population that may differ from the one usually included in cancer guidelines as multimorbidity is “the new normal” [19].

## Data Availability

Not applicable.
